# The music of the hemispheres: Cortical eigenmodes as a physical basis for large-scale brain activity and connectivity patterns

**DOI:** 10.3389/fnhum.2022.1062487

**Published:** 2022-11-24

**Authors:** Eli J. Müller, Brandon R. Munn, Kevin M. Aquino, James M. Shine, Peter A. Robinson

**Affiliations:** ^1^School of Physics, The University of Sydney, Sydney, NSW, Australia; ^2^Center for Integrative Brain Function, The University of Sydney, Sydney, NSW, Australia; ^3^Brain and Mind Center, The University of Sydney, Sydney, NSW, Australia

**Keywords:** eigenmodes, resting state networks, principal components, independent components, brain dynamics, brain connectivity

## Abstract

Neuroscience has had access to high-resolution recordings of large-scale cortical activity and structure for decades, but still lacks a generally adopted basis to analyze and interrelate results from different individuals and experiments. Here it is argued that the natural oscillatory modes of the cortex—cortical eigenmodes—provide a physically preferred framework for systematic comparisons across experimental conditions and imaging modalities. In this framework, eigenmodes are analogous to notes of a musical instrument, while commonly used statistical patterns parallel frequently played chords. This intuitive perspective avoids problems that often arise in neuroimaging analyses, and connects to underlying mechanisms of brain activity. We envisage this approach will lead to novel insights into whole-brain function, both in existing and prospective datasets, and facilitate a unification of empirical findings across presently disparate analysis paradigms and measurement modalities.

## 1. Introduction

Recent technological advances have seen a huge increase in data recorded from the brain, and in their spatial and temporal resolution, revealing striking complexity of neural activity up to whole-brain scales. In response, neuroscientists have attempted to compactly characterize these data, often decomposing signals into statistically derived components that maximize statistical independence, explained variance, or fidelity to anatomical and cytological features. Methods such as independent component analysis (ICA), principal component analysis (PCA), and clustering (McKeown and Sejnowski, 1998; Fischl et al., 2004; Desikan et al., 2006; Triarhou, 2007; Thomas Yeo et al., 2011; Abeysuriya and Robinson, 2016; Shine et al., 2019) typically produce 5–20 robust large-scale spatial patterns (Van De Ven et al., 2004; Damoiseaux et al., 2006) including the visual, attention, and default-mode ‘resting state networks’ (RSNs), and permit data classification and comparison between subjects and experiments. However, comparison between approaches and protocols is difficult, because of the lack of obvious compatibility of different experimental and data-processing choices, and most rely on ‘black-box’ statistical approaches that do not consider the sources or mechanisms behind the signals being analyzed. These factors limit their utility for understanding brain dynamics and one is motivated to seek a means to compactly represent large-scale brain activity and structure that is researcher- and protocol-independent, linked to physical mechanisms, and general enough to enable comparisons across different subjects and imaging modalities.

We argue that the natural oscillatory modes of the physical cortex (i.e., its spatial eigenmodes), analogous to the notes of a stringed instrument, represent the optimal basis set for the systematic decomposition of cortical neural activity. First, their physical interpretation as mutually independent ‘notes’ produced by the cortex provides an intuitive basis for understanding brain activity in a way that separates spatial and temporal structure. Furthermore, this basis provides a compact representation of neural dynamics with an ordering that is grounded in the physical structure of the brain and independent of stimuli. Second, if eigenmodes are the fundamental “notes” of the brain, one can then view the robust large-scale brain patterns identified by statistical means as akin to frequently played musical chords, each comprising a characteristic combination of notes. This viewpoint enables us to explain classical findings in whole-brain neuroimaging, such as the alternating engagement of the default-mode and attentional RSNs (Fox et al., 2005), as discussed below. Finally, eigenmodes provide insight into the structure of the cortex and how low-order modes can facilitate interareal communication in the absence of direct physical connection.

## 2. Notes of the cortex: Eigenmodes

**Figure 1:**
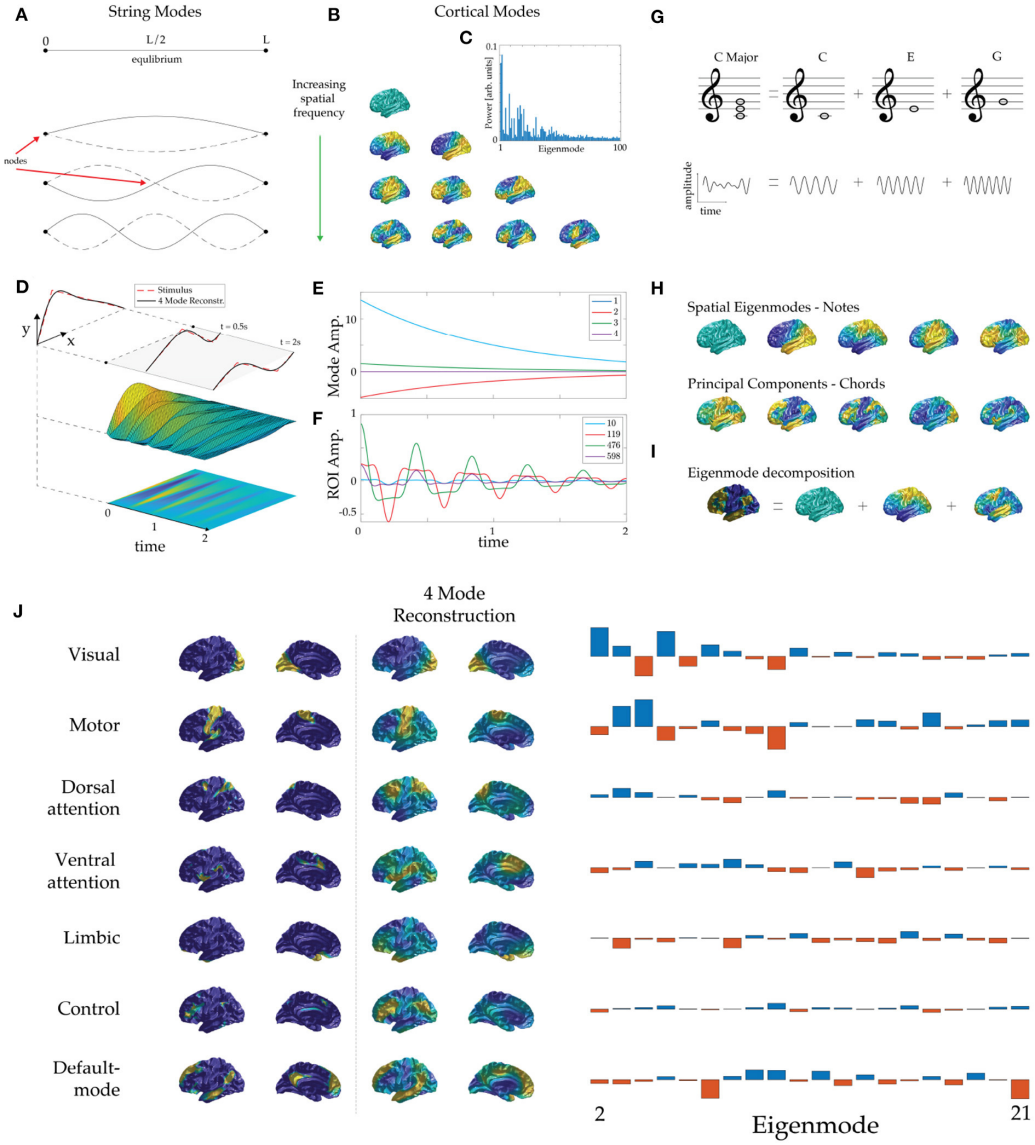
Eigenmode basis and dynamics. **(A)** Equilibrium position (top) and the lowest three eigenmodes of a violin string, ordered by increasing spatial frequency, showing zeros (nodes) and antinodes. (Note: we do not use “node” to denote an artificially discretized point on the cortex.) The solid dots indicate the clamped ends and solid and dashed curves show string positions half an oscillation apart in each case. **(B)** Lowest eigenmodes of an average cortical surface. Rows display cortical modes ordered by spatial frequency (more nodal lines) and warm and cool colors show positive and negative values relative to the mean at one extreme of an oscillation. **(C)** Human BOLD power (amplitude-squared) spectrum of eigenmodes during rest. The lower eigenmodes contribute the most power to ongoing neural activity. **(D)** Initial position of a plucked violin string and its mapping to the first four eigenmodes. **(E)** Subsequent evolution of mode amplitudes. **(F)** Subsequent evolution of displacement of several points along the violin string, analogous to regions-of-interest. **(G)** The C major chord is made up of a superposition of the notes C, E, and G, which have the frequency ratio 4:5:6; time series appear below. **(H)** Eigenmodes of the human cortex (top row) are analogous to “notes” of the cortex and statistically derived modes (bottom row, PCA modes here) are analogous to commonly recurring “chords” of cortical activity. **(I)** Analogously to music, cortical patterns can be decomposed into eigenmodes, whose amplitudes are its ‘fingerprint’. In this example we approximate Margulies's principal “gradient” pattern (chord) (Margulies et al., 2016) *via* its dominant constituent eigenmodes (notes). (The term gradient derives from the fact that it is calculated from quantities that have spatial gradients although it is not itself the gradient of any quantity.) **(J)** Eigenmode decomposition of the seven resting-state networks (RSNs) of Thomas Yeo et al. (2011). The first two columns show the RSNs formed by clustering correlations and weighted by the confidence of each point's attribution to its cluster to avoid spurious enhancement of high-order mode amplitudes by sharp edges. The third and fourth columns show each RSN reconstructed from its dominant four eigenmodes. The fifth column shows the modal amplitudes of each RSN, from which the dominant modes were identified.

The eigenmodes of a physical system typically comprise spatial patterns that oscillate at characteristic frequencies. For example, when a violin is plucked, or a drum is struck, natural frequencies are excited, each corresponding to a spatial pattern of displacement of the string or drumhead. Before being plucked, a violin string remains at rest in its equilibrium position, as seen in Figure 1A. Both ends of the string are fixed, so when it is plucked they remain stationary (they are termed zeros or nodes) while the rest of the string oscillates. The first three oscillatory modes are shown in Figure 1A, ordered by their number of zeros (and thus by spatial frequency). Significantly, each mode extends over the whole string and every point on the string is part of every mode. In the temporal domain, each spatial eigenmode generates a note whose frequency is determined by the string's physical properties, but constrained by its geometry because an integer number of half-wavelengths must fit exactly within its length.

Eigenmodes of any system are intrinsic to that system and are determined by its dynamics and geometry independently of any particular inputs or stimuli. Moreover, in a broad class of systems, eigenmodes are mutually independent and any arbitrary spatial pattern can be expressed as a weighted sum of eigenmodes. These properties make eigenmodes so useful that they have become ubiquitous throughout mathematics, science, and engineering, starting with Fourier's work more than 200 years ago (Fourier, 1822). Indeed, moving between coordinate-space and modal representations is essential to obtain maximal insight into almost any physical system.

In the case of the cortex, the closed cortical surface imposes a geometric constraint in two dimensions (2D) that determines the spatial structure of its eigenmodes. The resonant frequencies of brain rhythms are then set by a combination of this constraint and local dynamics, analogously to the case of the 1D violin string. Any pattern of brain excitation and structure can then be expressed in terms of these eigenmodes, including spontaneous and evoked brain activity (Nunez, 1989; Robinson et al., 2001; Gabay and Robinson, 2017; Mukta et al., 2020) and underlying brain connectivity (Robinson et al., 2014, 2016; Gao and Robinson, 2020).

The spatial structure of eigenmodes of the cortex (termed spatial eigenmodes for brevity) have been shown to be well approximated by assuming a governing wave equation and thus solving the Helmholtz equation on a cortical hemisphere (Nunez, 1989; Robinson et al., 2001; Pinotsis et al., 2013; Gabay and Robinson, 2017; Mukta et al., 2020).


(1) $$\nabla^{2}u(r)=-k^{2}u(r),$$

where **r** denotes spatial location. In this approximation, spatial eigenmodes *u*(**r**) of brain activity are eigenfunctions of the Laplace-Beltrami operator ∇^2^ with eigenvalues *k*^2^; this equation can be solved on cortical surfaces, such as ones estimated *via* MRI, using finite element methods (see Robinson et al., 2016; Gabay and Robinson, 2017 for mathematical details). Figure 1B shows examples of the spatial eigenmodes of an average cortical surface (Fischl, 2012).

As for other systems, cortical eigenmodes are mutually independent, so each provides independent spatial information. They are naturally ordered from low spatial frequency (globally uniform) to high spatial frequency (localized features), with the lowest modes having the longest-lived oscillations. When spontaneous or task-related activity with spatial structure given by a function *g*(**r**), where **r** is position, is decomposed into a sum over modes, the coefficient *c* of a mode *u* is given by the following integral over all **r** in the cortex:


(2) c=∫u(r)g(r)dr,

which is termed the *projection* of *g* onto *u*. The lowest modes are found to dominate the dynamics (Nunez, 1989; Robinson et al., 2001; Wingeier et al., 2001; Mukta et al., 2020), as illustrated by the power spectrum of human blood-oxygen-level-dependent (BOLD) activity during rest in Figure 1C. This explains why only 5–20 robust spatial patterns are identified by statistical means. A useful feature of Equation (2) is that it integrates over short-scale noise, and hence tends to suppress it, thereby removing the main motivation for thresholding.

When stimuli enter the brain (or when a violin string is plucked), eigenmodes are excited with initial amplitudes given by Eq. (2), with *g*(**r**) representing the initial stimulus. These amplitudes then decay at the damping rates appropriate to each mode (Mukta et al., 2020). Figure 1D illustrates the dynamics of a violin string that is plucked (i.e., release from an initial triangular shape) and its approximation by just the lowest four nonzero eigenmodes. We see that this provides a good approximation of the shape of the string, both then and at later times. Additionally, the subsequent temporal dynamics of the string is described by exponentially decreasing mode amplitudes, as shown in Figure 1E. In contrast, the time evolution of displacements of various points along the violin string—an analog of region-of-interest (ROI) time-series—gives a more complicated and obscure picture, as seen in Figure 1F. This result highlights the benefits of representing complex brain dynamics *via* its spatial eigenmodes and we expect these representations will expand functional insights.

## 3. Music of the cortex: Notes and chords

Music involves vibrational modes of instruments, excited at various frequencies and times. A chord such as C major has a complex periodic waveform that comprises superposed sine waves at the frequencies of the individual notes C, E, and G, as shown in Figure 1G. An electronic synthesizer constructs chords in just this way, but a musician plays chords directly, rather than exciting individual sine waves. Some chords are very common in particular pieces of music and thus may be more easily detected in statistical analyses than less common isolated notes. Each chord has a unique temporal signature but shares notes with other chords, establishing a fundamental interdependence. Hence, while chords provide a useful and efficient way to capture recurring musical motifs, an understanding of the underlying notes is essential to facilitate comparisons and groupings of chord families and links to the mechanisms by which instruments generate sound.

The above points lead to a direct analogy with the brain: if cortical eigenmodes correspond to its notes [Figure 1H (top)] then large-scale statistically detected patterns of recurrent brain activity can be viewed as its chords [Figure 1H (bottom)]. Frequently recurring patterns likely emerge from similar ‘plucking’ *via* related external stimuli or endogenous changes (e.g., large-scale neuromodulation). This view is consistent with the pervasive visual (sensory) and attentional (neuromodulatory) patterns seen in whole-brain imaging data (Thomas Yeo et al., 2011).

Any cortical pattern can be uniquely decomposed into eigenmodes, as illustrated in Figure 1I. Figure 1J (left) shows the seven widely cited RSNs of Yeo et al., where the confidence of each region's attribution to a particular RSN has been used to spatially smooth the patterns to remove artifactual sharp edges (Thomas Yeo et al., 2011). The reconstruction of each RSN using the four dominant (highest amplitude) eigenmodes in each case is shown, reflecting differing combinations of eigenmodes [Figure 1J (right)]. Interestingly, the default mode RSN and the dorsal attention RSN project with opposite sign onto the dominant low spatial frequency modes (eigenmodes 2–4; i.e., those with a single nodal line), so when any of these three eigenmodes oscillates, the default-mode and the dorsal-attention RSNs will oscillate 180° out of phase, thus providing a simple mechanistic explanation for the finding that these RSNs are temporally anticorrelated (Greicius et al., 2003; Fox et al., 2005).

We expect that eigenmode analysis will facilitate further such mechanistic insights into patterns of whole brain activity detected *via* various imaging methods (Atasoy et al., 2016). Furthermore, eigenmodes may explain a similar axis of separation that has been demonstrated in functional-MRI data using diffusion embedding (Margulies et al., 2016). Follow-up work by Raut et al. (2021) has also shown that a very similar spatial pattern is found in the oscillatory phase-shifts observed relative to subject arousal levels measured *via* respiratory variation. This phase relationship may be mechanistically interpreted in terms of physical eigenmodes; i.e., arousal is coincident with the promotion of a particular family of oscillatory eigenmodes.

## 4. Modal communication channels

**Figure 2:**
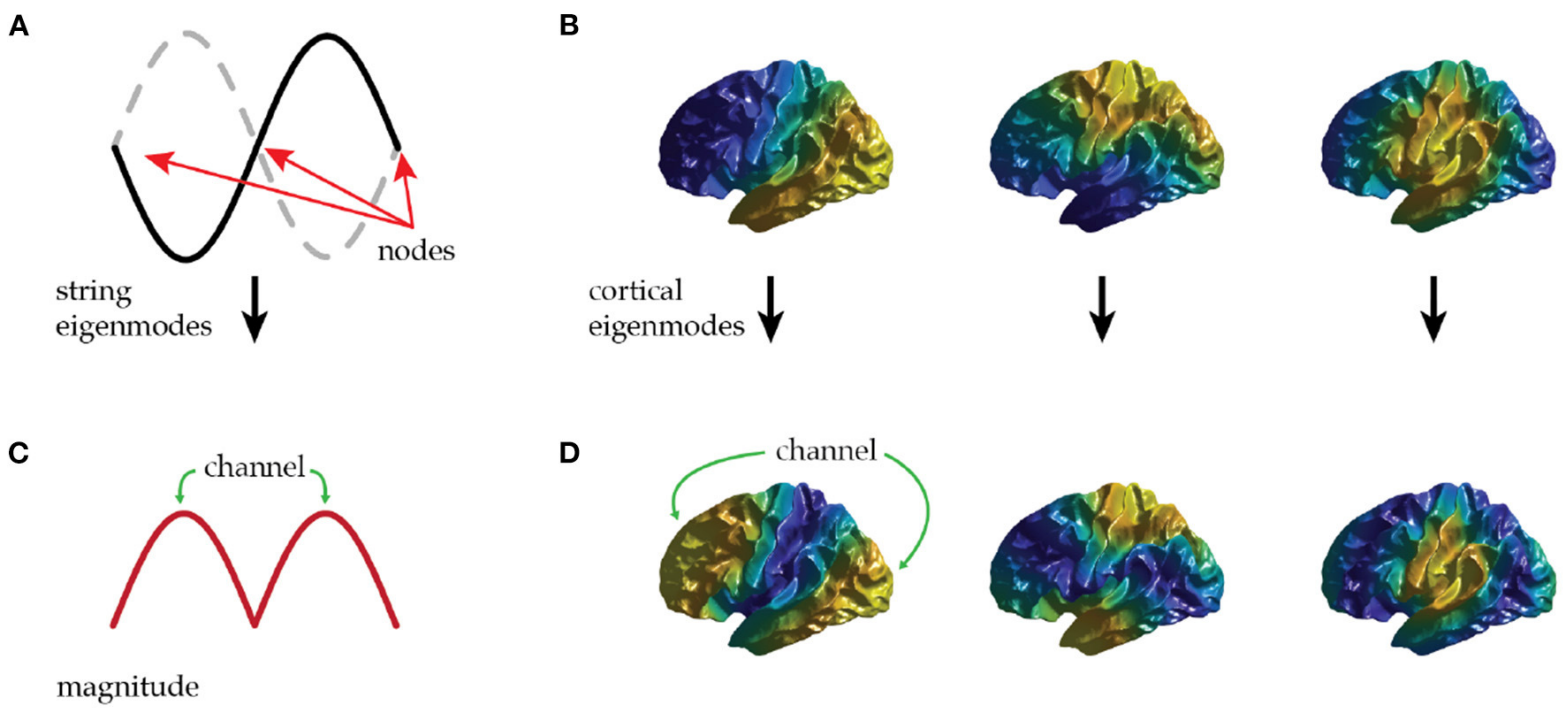
**(A)** The first non-uniform eigenmode of a string contains three zeros (nodes) and two antinodes that oscillate in antiphase. **(B)** The first three non-uniform eigenmodes of the cortex—each has a single nodal line. **(C)** The local amplitude (magnitude) of the first non-uniform eigenmode of a string shows a possible communication channel between each antinode. **(D)** The local amplitude of the first three non-uniform cortical eigenmodes supports communication along rostrocaudal, dorsoventral, and mediolateral axes.

Spatial eigenmodes offer a potential channel to mediate communication between distant brain regions. Equation (2) shows that modes are most easily excited where |*u*(**r**)| is largest; i.e., at antinodes; likewise, their influence on local activity is largest at antinodes. Hence, each mode provides a channel for preferential communication between regions centered on its antinodes, as illustrated in Figures 2A,C for the second mode of a violin string.

Applying this idea to the lowest cortical modes, we see that the first (uniform) mode mediates communication approximately equally between all regions of the cortex, thus providing a means for any region to access the typical level of excitation of the brain as a whole. The next three modes have a single nodal line each with a pair of antinodes. Figures 2B,D shows that these are aligned along the rostrocaudal axis, the dorsoventral axis, and the mediolateral axis of the brain hemisphere. Each of these modes can thus preferentially mediate communication along one of these principal axes. Oscillatory activity transmitted in this way may provide a large-scale analog of the communication-through-coherence mechanism originally introduced at short scales by invoking roughly 40 Hz gamma oscillations to preferentially excite responses at particular phases (Fries, 2005). Here we argue that modal oscillations could also enhance responses at particular spatial locations defined by the antinodes of spatial cortical eigenmodes.

To illustrate these points, consider the first mode in Figure 2B with rostro-caudal orientation. This mode's antinodes are in prefrontal cortex and posterior sensory cortex, with a nodal line running through somatomotor cortex. This provides a communication channel between sensory and prefrontal regions that only weakly involves intermediate zones. These intermediate zones thus interact little with this mode. The analogy with a violin string is helpful in seeing that this is not problematic, the second mode seen in Figure 1A peaks at two points (Figure 1C) with a zero at the center, despite the string being continuous through the zeros.

Neural activity is dominated by only a few low order eigenmodes, as shown in Figure 1C. These modes are continuous, accessible everywhere in the brain, and integrate over fine scale structure and inputs. We thus speculate these dominant modes play an important role in supporting cognition and states of consciousness by providing channels for communication between distal cortical regions that do not necessarily possess direct physical connections.

Finally, the spatial patterns of the eigenmodes suggest a novel stimulation strategy to effectively and deliberately manipulate large-scale cortical activity— plucking a violin string near antinodes of a given eigenmode will have the greatest impact on the amplitude of that eigenmode. This suggests that systems for measurement or stimulation could usefully exploit eigenmode structures—particularly those of the low-order dominant eigenmodes. Indeed, key features of the empirically observed evoked response of the brain to spatially localized impulse stimuli are found to be well described by only a few eigenmodes (Mukta et al., 2020). This insight is relevant for transcranial magnetic stimulation and other stimulation technologies used to probe cognition and treat pathologies. The power of systems that can excite low level eigenmodes may also help to explain how small but widely projecting neuromodulatory sources, such as the adrenergic locus coeruleus and nucleus basalis of Meynert (Shine et al., 2021; Wainstein et al., 2022), can have a large effect on cortical dynamics. We suggest that future experiments investigate how the neuromodulatory systems interact with cortical eigenmodes and that this may assist in optimizing cortical stimulation protocols.

## 5. Concluding remarks

In this manuscript we have outlined various advantages of a cortical eigenmode basis of the brain:

(i) Eigenmodes satisfy the main criteria for an optimal basis set in that they are readily interpretable and leverage the intuitive understanding of natural resonances or notes of the cortex—equivalent to notes of a string.
(ii) A key goal of neuroscience is to unify theories of brain activity, function, and structure. First and foremost this requires recordings and analysis of brain activity to be generalizable and thus comparable across recording sessions, different tasks, subjects, and measurement methods. In neuroimaging this has been approached *via* “resting-state networks” and popular parcellation schemes (Thomas Yeo et al., 2011; Gordon et al., 2016; Schaefer et al., 2018); however, as we discussed in previous sections, these are often constructed *via ad hoc* statistical measures, which limits interpretability and prevents standardization. In the worst case, each new approach requires the research community to establish mutual interpretability between it and all others—an overall burden that scales as the square of the total number of methods in use. However, much as English often serves as a common language through which other languages can be translated, eigenmodes provide a route by which only a single extra interpreter is required for each new method (or language, analogously) added. In other words, cortical eigenmodes can serve as a common basis through which to interrelate new findings and existing knowledge.
(iii) Eigenmodes are easily generalizable, independent of stimuli and experimental choices, and result from the brain's structure—avoiding the artificial warping and thresholding required for analyses *via* parcellations and artificially discretized networks. As such, eigenmodes remain applicable regardless of future improvements in resolution and accuracy of brain measurements and imaging.
(iv) Eigenmodes provide insight into the whole-brain function with parallel communication channels possible between cortical areas with no direct physical connection.
(v) Eigenmodes offer a simple explanation to the perplexing finding that the dorsal attention network and default mode network are anticorrelated. And further a cortical pattern revealed in fMRI data separating primary sensory and association areas, which has been recapitulated in oscillatory phase shifts tied to subject arousal, can be simply interpreted as an arousal evoked family of oscillatory cortical eigenmodes.

The eigenmodes in this work are considered purely for the cortex, and an identical set exists for each hemisphere. This presents an exciting opportunity to extend eigenmode analysis to subcortical loci including key structures such as the thalamus, hippocampus, and cerebellum.

The above advantages favor the wider adoption of eigenmodes in neuroscience that will provide both theoretical and empirical insight, as it has done for the fields of physics, mathematics, and engineering, thereby opening up exciting opportunities for future work.

## Data availability statement

The original contributions presented in the study are included in the article/supplementary material, further inquiries can be directed to the corresponding author.

## Author contributions

EM and BM contributed equally to all facets of this project. KA, JS, and PR contributed to concepts and writing of the manuscript. All authors contributed to the article and approved the submitted version.

## Funding

This work was supported by the Australian National Health and Medical Research Council *via* Grant GNT1193857 and by the Australian Research Council Center of Excellence Grant CE140100007, and the Australian Research Council Laureate Fellowship Grant FL140100025.

## Conflict of interest

The authors declare that the research was conducted in the absence of any commercial or financial relationships that could be construed as a potential conflict of interest.

## Publisher's note

All claims expressed in this article are solely those of the authors and do not necessarily represent those of their affiliated organizations, or those of the publisher, the editors and the reviewers. Any product that may be evaluated in this article, or claim that may be made by its manufacturer, is not guaranteed or endorsed by the publisher.
